# EarlyPostoperative Lactate-to-Preoperative Albumin Ratio with In-Hospital Mortality After Elective Colorectal Cancer Surgery: A Single-Center Retrospective Cohort Study

**DOI:** 10.3390/jcm15166404

**Published:** 2026-08-19

**Authors:** Orhan Aslan, Mehmet Oğuzhan Polat, Aşkın Kadir Perçem, Ramazan Topcu, Mahmut Arif Yüksek, Mustafa Şahin

**Affiliations:** 1 Department of General Surgery, Faculty of Medicine, Hitit University, 19040 Çorum, Turkey; oguzhanmehm@gmail.com (M.O.P.); topcur58@gmail.com (R.T.);; 2 Izmir Tepecik Training and Research Hospital, 35170 Izmir, Turkey; 3 Department of Biochemistry, Faculty of Medicine, Hitit University, 19240 Çorum, Turkey; mustafa.sahin57@saglik.gov.tr

**Keywords:** biomarkers, colorectal cancer, hospital mortality, lactate-to-albumin ratio, postoperative period

## Abstract

**Background**: Risk stratification after colorectal cancer surgery remains challenging. We evaluated whether a perioperative ratio combining immediate postoperative lactate with preoperative albumin is associated with in-hospital mortality after elective colorectal resection. **Methods**: In this single-center retrospective cohort, 282 patients underwent open elective colorectal resection. The perioperative lactate-to-albumin ratio (LAR) was calculated as arterial lactate (mmol/L) divided by serum albumin (g/dL), and discrimination was assessed by receiver operating characteristic (ROC) analysis with age-adjusted association by Firth’s penalized logistic regression and fixed-model bootstrap validation. **Results**: Seventeen patients (6.0%) died in hospital, and mortality rose across LAR tertiles (2.1%, 5.3%, and 10.6%; *p* = 0.014). LAR showed moderate discrimination (AUC 0.73; 95% CI 0.58–0.87; optimism-corrected AUC 0.78), with no evidence of better discrimination than lactate or albumin alone. At the Youden threshold of 0.555, sensitivity was 76.5% and specificity 61.1%. The age-adjusted Firth odds ratio was 1.19 per 0.1-unit increase (95% CI 1.09–1.29). **Conclusions**: The perioperative lactate-to-albumin ratio was associated with in-hospital mortality after age adjustment in this single-center cohort. Given the small number of deaths and absence of external validation, LAR should be regarded as a candidate marker requiring prospective multicenter validation before clinical application.

## 1. Introduction

Despite advances in perioperative care, early complications and mortality remain important after colorectal cancer surgery, particularly in older adults [1,2]. Preoperative tools such as the American College of Surgeons National Surgical Quality Improvement Program risk calculator estimate baseline procedural risk, while preoperative albumin provides information on inflammatory and nutritional vulnerability [3,4]. These assessments support planning but cannot fully capture the physiological response to the operation.

Postoperative risk is shaped by the magnitude of surgical stress, hemodynamic disturbance, complications, and perioperative care. Enhanced Recovery After Surgery (ERAS) pathways standardize multiple elements of colorectal perioperative management, and randomized evidence indicates that laparoscopic and open approaches can produce different inflammatory responses [5,6]. These factors can influence postoperative biomarkers and should be considered when interpreting any single laboratory-derived risk marker.

In this study, lactate and albumin represent different perioperative time points. Albumin was the last preoperative value, usually measured within 24 h before surgery, whereas lactate was the first arterial blood-gas value obtained within 1 h after intensive care unit admission. The resulting ratio therefore combines baseline reserve with the immediate metabolic response to surgery; it is not a postoperative LAR in which both components are measured after surgery. LAR has shown prognostic value in several critical-care settings [7,8], the lactate dehydrogenase-to-albumin ratio has been studied after colorectal cancer surgery [9], and early postoperative arterial lactate has been associated with outcomes after major surgery [10]. Whether this mixed-time-point perioperative LAR is associated with in-hospital mortality after elective oncologic colorectal resection remains uncertain.

The aim of this study was to evaluate the discrimination and age-adjusted association of immediate postoperative lactate-to-preoperative albumin ratio with in-hospital mortality after elective colorectal cancer surgery. Given the small number of deaths, all predictive findings and the data-derived cutoff were considered exploratory and hypothesis-generating.

## 2. Materials and Methods

### Patient Selection and Study Design

**Figure 1:**
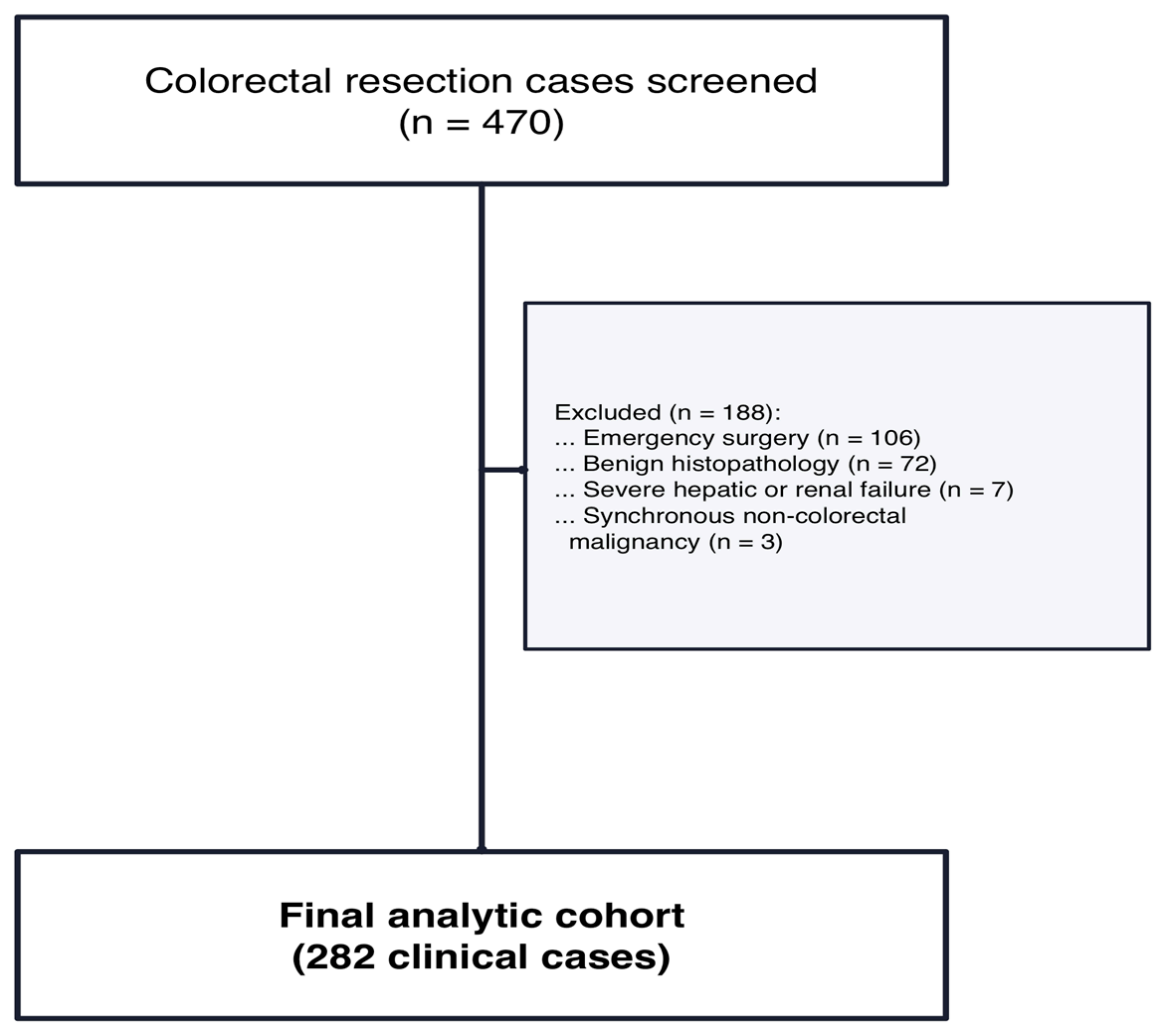
Patient selection flowchart. The final analytic cohort contained 282 patients with preoperative albumin and immediate postoperative lactate values.

This retrospective cohort study was conducted in the Department of General Surgery, Hitit University Erol Olçok Training and Research Hospital, Çorum, Turkey, with approval from the Hitit University Faculty of Medicine Research Ethics Committee (decision no. 2025-169). Consecutive colorectal resection records from January 2018 through August 2025 were screened. Screening identified 470 surgical cases, of which 188 were excluded because of emergency surgery (*n* = 106), benign histopathology (*n* = 72), severe hepatic or renal failure (*n* = 7), or a synchronous non-colorectal malignancy (*n* = 3), leaving 282 eligible patients. Throughout the study period, perioperative care followed the center’s standard institutional practice. No formal Enhanced Recovery After Surgery (ERAS) protocol was implemented, and no structured ERAS adherence was recorded for any patient. The cohort therefore reflects conventional perioperative management, which should be considered when comparing these results with those from centers operating audited ERAS pathways. The study is reported in accordance with STROBE. The case-selection flowchart is shown in Figure 1.

Inclusion Criteria: Age 18 years or older; elective colorectal resection; histopathologically confirmed colorectal adenocarcinoma; a last preoperative albumin measurement; and a first arterial blood-gas lactate measurement obtained within 1 h after intensive care unit admission.

Exclusion Criteria: Emergency surgery, benign histopathology, severe hepatic or renal failure, and synchronous malignancy other than colorectal cancer.

Data Collection: Patient data were retrieved from the hospital information management system. Age, sex, comorbidities, tumor localization and stage, neoadjuvant therapy, operation duration, preoperative laboratory values, the first intensive care arterial blood-gas lactate, and clinical outcomes were recorded. Tumor stage was classified using the tumor-node-metastasis system. Postoperative bleeding was defined as bleeding requiring reoperation or transfusion. Anastomotic leak was defined as clinically or radiologically confirmed disruption of the anastomosis. Surgical site infection was classified according to Centers for Disease Control and Prevention criteria. All resections were performed as open procedures; no laparoscopic, robotic, or converted operations were included. Every patient was admitted to the intensive care unit immediately after surgery under routine institutional policy rather than because of intraoperative deterioration. Intensive care and total hospital length of stay were counted in calendar days, so a patient transferred to the ward before midnight on the day of surgery is recorded as zero intensive care days.

- Demographic data (age, sex).
- Clinical features (comorbidities, tumor localization and stage).
- Operative data (operation duration).
- Laboratory findings (preoperative WBC, CRP, albumin, and total protein; immediate postoperative lactate).
- Clinical outcomes: In-hospital mortality (death during the index hospitalization), postoperative bleeding (requiring reoperation or transfusion), anastomotic leak (confirmed clinically or radiologically), surgical site infection, intensive care unit (ICU) length of stay (days), and total hospital length of stay (days).

LAR Calculation and Sampling Time: The numerator was the first lactate concentration (mmol/L) measured from an arterial blood-gas sample obtained within 1 h after intensive care unit admission. Because every patient was admitted to intensive care after surgery and arterial blood-gas analysis within the first hour of that admission is routine institutional practice, the sample was obtained as part of standard postoperative care rather than in response to clinical deterioration, and no venous samples were used. The denominator was the last preoperative serum albumin concentration, typically obtained within 24 h before surgery. Albumin stored as g/L was converted to g/dL before calculation. LAR was calculated as postoperative lactate (mmol/L) divided by preoperative albumin (g/dL) and is reported in conventional mixed units (mmol/L)/(g/dL). Values would change tenfold if albumin were entered in g/L without conversion. The ratio combines two different perioperative time points.

Statistical Analysis: Continuous variables were assessed with the Shapiro–Wilk test and are reported as mean ± SD or median [IQR]. Given only 17 deaths, LAR tertiles were used descriptively to retain balanced groups and reduce sparse outcome cells; continuous LAR was the primary regression exposure. Group comparisons used one-way ANOVA or Kruskal–Wallis tests for continuous variables and Pearson chi-square or Fisher–Freeman–Halton tests for categorical variables. Cochran–Armitage tests assessed trends in mortality and bleeding. Because every resection was open, the surgical approach was constant and could not be evaluated as a variable; as an exploratory analysis of anatomical variation, and not as a substitute for procedure type, LAR was compared across tumor localization groups by Kruskal–Wallis test and mortality by Fisher–Freeman–Halton test. Secondary outcomes were exploratory and were not adjusted for multiple testing. ROC analysis quantified discrimination. The Youden index identified a data-derived threshold, and diagnostic measures were calculated from the corresponding 2 × 2 table. Correlated ROC curves were compared with paired DeLong tests. Given the small event count, the primary multivariable Firth’s penalized logistic model was deliberately restricted to continuous LAR and age, selected for clinical relevance. Other recorded variables were examined only in exploratory univariable analyses; no significance-based stepwise selection defined the primary model. The unrounded ROC threshold was used in a separate exploratory age-adjusted dichotomous model. Fixed-model internal validation used 1000 bootstrap resamples with seed 2025; optimism in AUC was estimated by fitting the same age-plus-LAR Firth model in every resample. A three-knot restricted cubic spline at the 10th, 50th, and 90th percentiles was an exploratory standard-logistic analysis of dose–response shape. Analyses used R 4.5.2. Two-sided *p* < 0.05 was used without implying clinical importance.

## 3. Results

### 3.1. Baseline Characteristics

A total of 282 patients were stratified into LAR tertiles (*n* = 94 per group). Seventeen deaths (6.0%) occurred during the index hospitalization. Preoperative inflammatory and nutritional markers differed across ascending tertiles, as did immediate postoperative lactate (Table 1).

### 3.2. Outcomes Across LAR Tertiles

In-hospital mortality increased across ascending LAR tertiles (2.1%, 5.3%, and 10.6%; Cochran–Armitage trend *p* = 0.014), as did postoperative bleeding (1.1%, 2.1%, and 7.4%; *p* = 0.018). Intensive care unit stay differed across tertiles (Kruskal–Wallis *p* = 0.009; ε^2^ = 0.033), although the effect size was small. These secondary findings are exploratory, and the temporal relation between immediate lactate and early bleeding may be bidirectional. Other outcomes did not show clear differences (Table 1). Every resection was open, so surgical approach did not vary; in an exploratory localization analysis, neither LAR (Kruskal–Wallis *p* = 0.16) nor mortality (Fisher–Freeman–Halton *p* = 0.71) differed across tumor localization groups, although these comparisons were underpowered given 17 deaths and sparse categories.

### 3.3. Discrimination for In-Hospital Mortality

**Figure 2:**
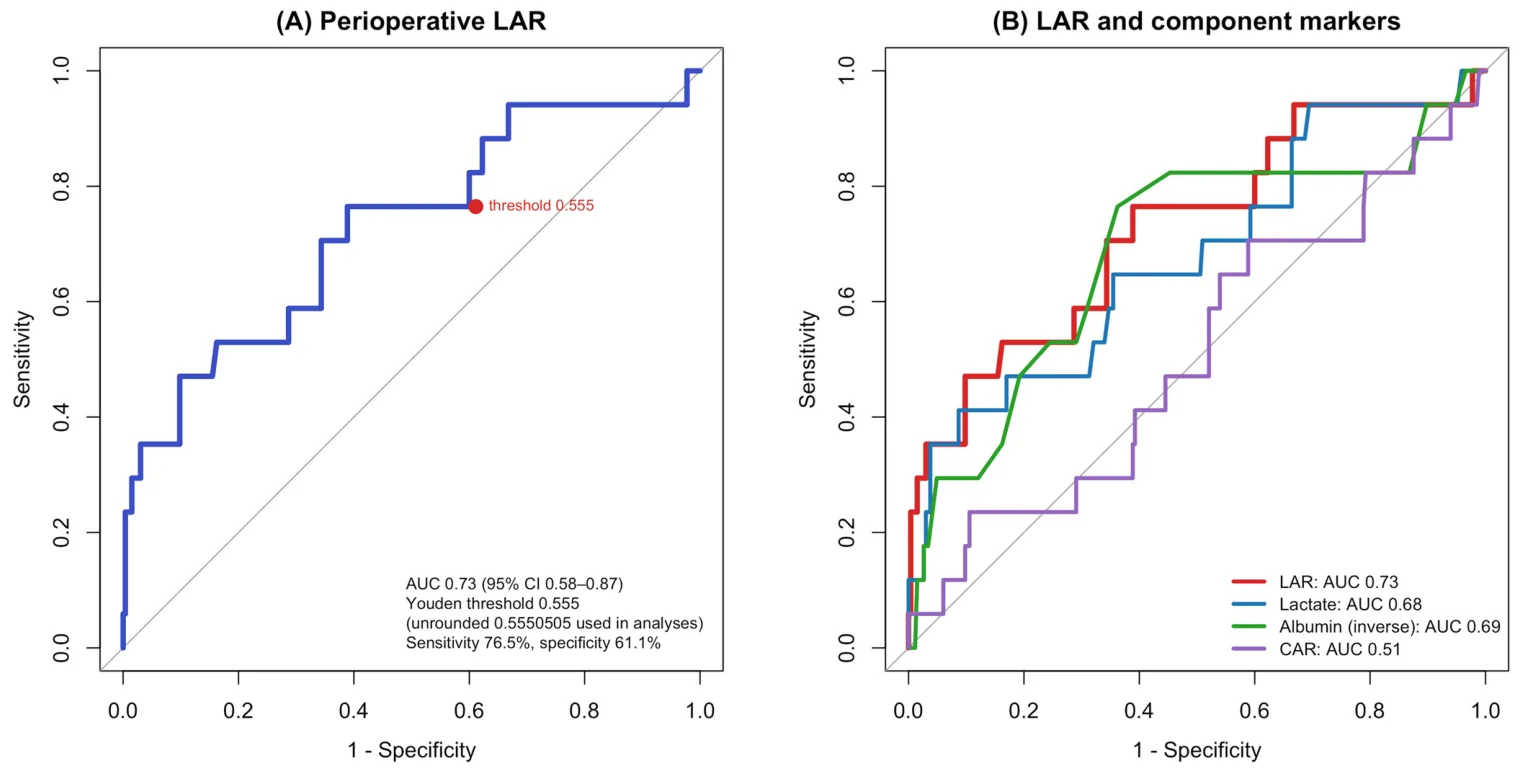
Receiver operating characteristic curves for in-hospital mortality. (**A**) Perioperative LAR alone; AUC 0.73 (95% CI 0.58–0.87), with the Youden operating point marked (threshold 0.555; the unrounded value 0.5550505 was used in analyses). (**B**) The same LAR curve compared with immediate postoperative lactate, preoperative albumin (inverse orientation), and the preoperative CRP-to-albumin ratio. In both panels the diagonal grey line is the reference line of no discrimination (AUC 0.50).

LAR showed moderate apparent discrimination with substantial uncertainty for in-hospital mortality (AUC 0.73; 95% CI 0.58–0.87; *p* = 0.002). The unrounded Youden threshold was 0.5550505. At this threshold, sensitivity was 76.5% (exact 95% CI 50.1–93.2), specificity 61.1% (55.0–67.0), positive predictive value 11.2% (6.1–18.4), and negative predictive value 97.6% (93.9–99.3); positive and negative likelihood ratios were 1.97 and 0.38. The high negative predictive value is strongly influenced by the 6.0% mortality prevalence, while the low positive predictive value limits stand-alone rule-in use. Because the threshold was derived and evaluated in the same cohort, all performance estimates are optimistic and exploratory (Figure 2A).

### 3.4. Comparison with Component and Competing Markers

LAR showed no evidence of better discrimination than lactate (AUC 0.73 vs. 0.68; ΔAUC 0.049; 95% CI −0.014 to 0.112; paired DeLong *p* = 0.126) or albumin in inverse orientation (0.73 vs. 0.69; ΔAUC 0.038; 95% CI −0.103 to 0.179; *p* = 0.597). Non-significant differences do not establish equivalence. LAR discriminated better than CAR in this cohort (0.73 vs. 0.51; ΔAUC 0.217; 95% CI 0.044–0.390; *p* = 0.014), but CAR itself performed no better than chance (Figure 2B).

### 3.5. Independent Association with In-Hospital Mortality

**Figure 3:**
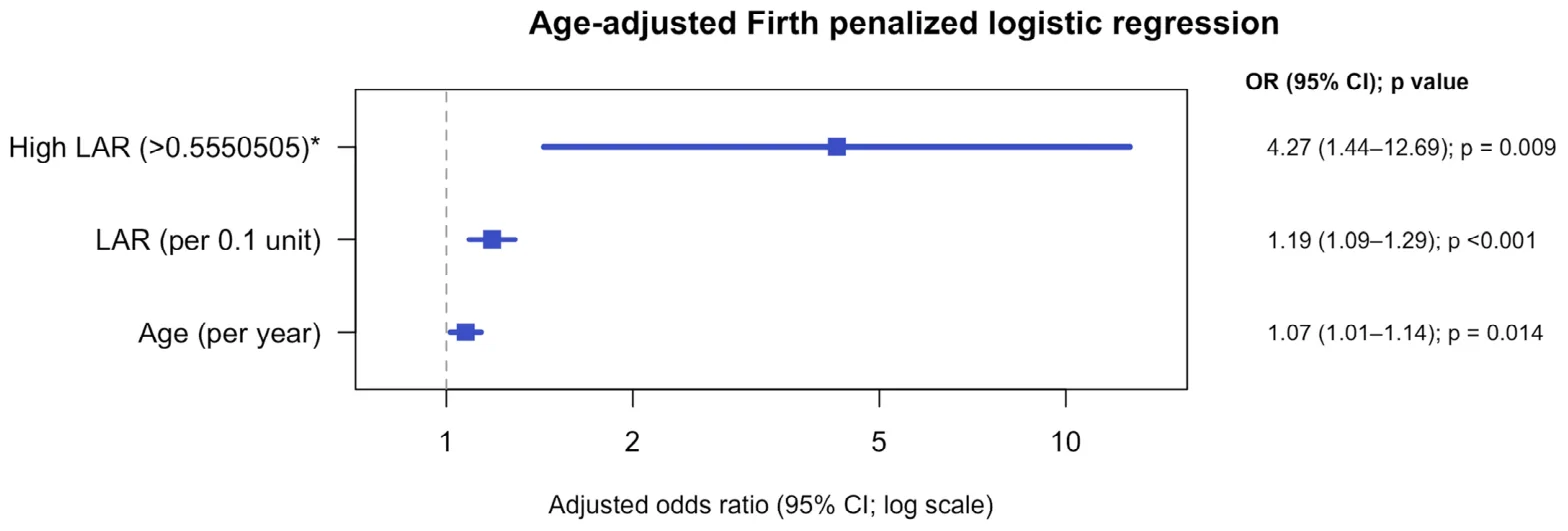
Age-adjusted Firth penalized logistic regression estimates for in-hospital mortality. Squares indicate adjusted odds ratios (ORs), horizontal lines indicate 95% confidence intervals (CIs), and dashed vertical line indicates null value (OR = 1). * The high-LAR estimate was obtained from a separate exploratory age-adjusted model using unrounded Youden threshold (>0.5550505); continuous and dichotomized LAR were not entered together. LAR, lac-tate-to-albumin ratio.

Exploratory univariable Firth models are shown in Table 2. The primary multivariable model included only age and continuous LAR because 17 deaths did not support a larger stable model. LAR remained associated with mortality after age adjustment (OR 1.19 per 0.1-unit increase; 95% CI 1.09–1.29; *p* < 0.001), and age was also associated with mortality (OR 1.07 per year; 95% CI 1.01–1.14; *p* = 0.014). Apparent model AUC was 0.79; fixed-model bootstrap validation yielded an optimism-corrected AUC of 0.78. In a separate exploratory model using the unrounded ROC threshold, high LAR (>0.5550505) had an age-adjusted OR of 4.27 (95% CI 1.44–12.69; *p* = 0.009). These estimates are imprecise and hypothesis-generating (Table 2, Figure 3).

### 3.6. Dose–Response Relationship

**Figure 4:**
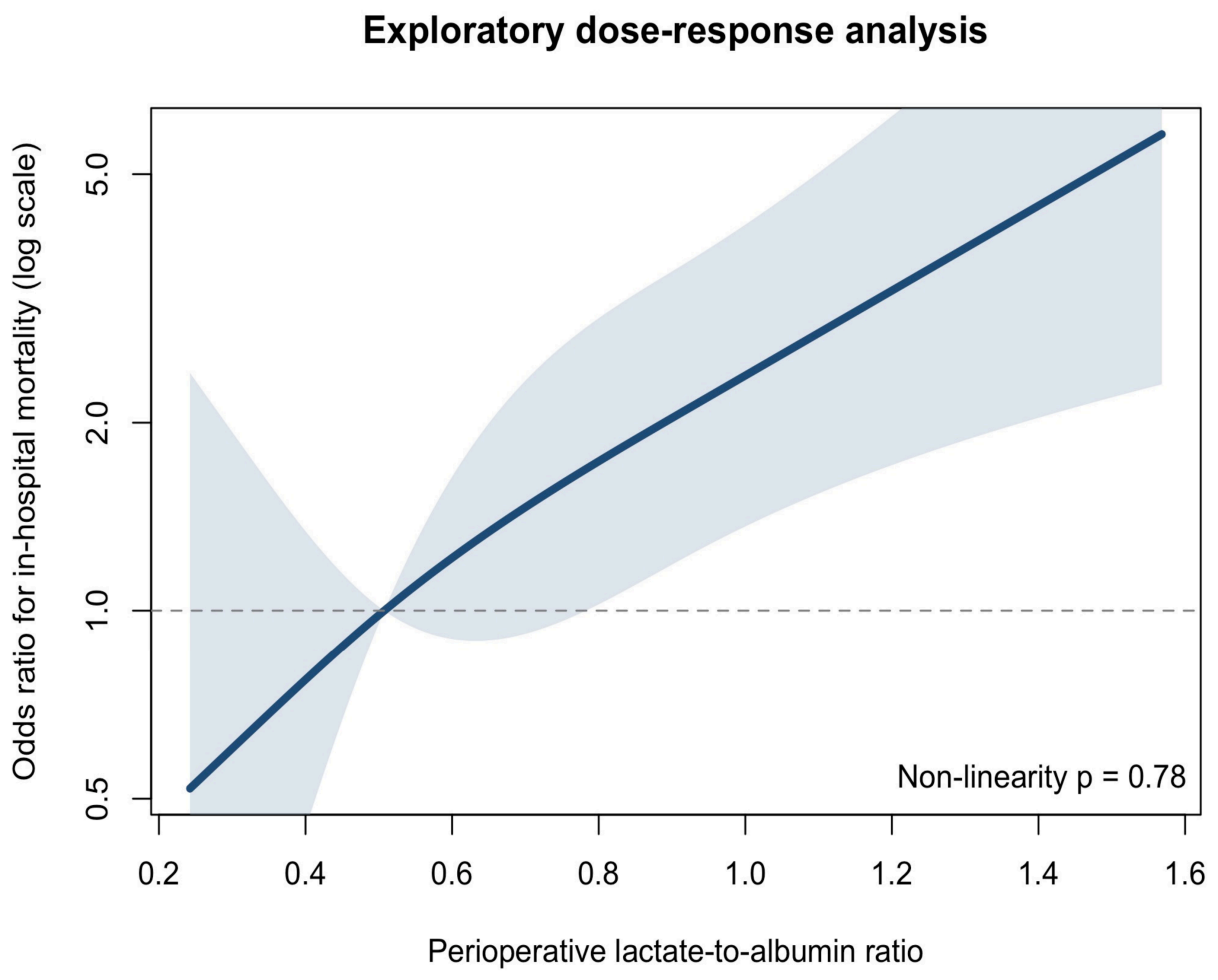
Exploratory restricted cubic spline for perioperative LAR and in-hospital mortality using standard logistic regression and three knots. Shading shows 95% CI; wide intervals reflect sparse events.

In an exploratory unadjusted three-knot restricted cubic spline, the fitted odds increased across observed LAR values, but confidence intervals were wide. There was no evidence of departure from linearity (*p* = 0.78); with only 17 deaths, this test had limited power and did not validate linearity (Figure 4).

## 4. Discussion

The principal finding was an age-adjusted association between higher perioperative LAR and in-hospital mortality. Mortality increased across descriptive tertiles, and the continuous age-adjusted Firth estimate was 1.19 per 0.1-unit increase. Because only 17 deaths occurred and key perioperative confounders were unavailable, the result is best considered hypothesis-generating rather than an independent biological or causal effect.

Apparent discrimination was moderate (AUC 0.73; 95% CI 0.58–0.87), with substantial uncertainty. LAR showed no evidence of better discrimination than lactate or albumin alone; failure to detect a difference does not prove equivalence. The ratio therefore has no demonstrated incremental discriminative value over its components in this cohort. Its apparent advantage over CAR mainly reflects that CAR performed near chance.

Immediate postoperative lactate can reflect hypoperfusion, catecholamine-driven metabolism, impaired hepatic clearance, operative stress, and early resuscitation [11]. Higher early postoperative lactate has been associated with adverse outcomes after major abdominal surgery [12,13,14]. Preoperative albumin reflects inflammatory burden and physiological reserve but is not a specific measure of nutrition [15]. Our perioperative LAR combines these distinct signals across two time points; it should not be interpreted as a simultaneous biochemical measurement or as evidence of a single causal pathway.

Preoperative risk calculators, baseline albumin, operative factors, and perioperative care pathways address different parts of perioperative risk [3,4,5,6]. The present ratio cannot replace these assessments. Instead, it tests whether immediate lactate, scaled to preoperative albumin, is associated with outcome after surgery. Surgical approach was uniform because every resection was open, which removes approach as a source of confounding but also confines these findings to open surgery; ASA status, frailty, and intraoperative blood loss were unavailable, so their influence on the observed association could not be quantified. Perioperative care at the study center followed conventional practice without a formal ERAS protocol. Because minimally invasive resection attenuates the inflammatory response [6] and ERAS pathways alter fluid management, feeding, and mobilization, both change the physiological trajectory that early lactate reflects, so these findings should not be assumed to transfer unchanged to laparoscopic or robotic cohorts or to centers with audited ERAS programs.

Prior studies have reported prognostic associations for LAR in critical-care populations [16,17,18]. Direct comparison of their thresholds with ours is limited because our numerator was immediate postoperative lactate and our denominator was preoperative albumin. Differences in sampling time, albumin units, case mix, resuscitation, and mortality prevalence can materially change both the ratio and its optimal cutoff. Specimen type is a further source of variation that is rarely reported. Single-center surgical and trauma studies that state the specimen have used arterial blood gas [19,20], whereas large database analyses using MIMIC-IV or eICU derived LAR from lactate and albumin measurements obtained within 24 h, without reporting arterial versus venous sampling [21,22]; the MIMIC-IV study explicitly selected the first available values [22]. Because arterial and peripheral venous lactate may not be interchangeable with respect to absolute values, pooling specimen types could shift the derived ratio and its estimated threshold [23]. The protocol-defined arterial sample within 1 h of intensive care admission used here narrows this source of variation relative to 24 h windows of mixed specimen type, but it does not eliminate it.

Evidence in surgical oncology remains limited. Reports in critically ill patients after open gastric cancer surgery [19], in patients with postoperative intestinal obstruction [21], and in mixed trauma and surgical intensive care cohorts [22] suggest that LAR may identify higher-risk patients, and serial lactate has been evaluated after gastrointestinal perforation [24]. One of these merits direct comparison, because it used the same exposure construction as ours after open cancer resection: preoperative albumin, taken to represent baseline reserve, divided into the first arterial blood-gas lactate obtained within an hour of postoperative intensive care admission, taken to represent acute stress [19]. In that cohort, LAR discriminated 28-day mortality significantly better than lactate (DeLong *p* = 0.006) and albumin (*p* = 0.043) and remained independently associated with death after adjustment, whereas we found no discrimination gain over either component. The exposure is shared, but the populations are not, and the differences may partly explain the divergence. That study enrolled gastrectomy patients admitted to intensive care specifically because of postoperative haemodynamic instability requiring vasopressor support; mean albumin was 2.80 g/dL and 24.5% died within 28 days. Ours were elective colorectal resections in which every patient entered intensive care by routine policy rather than by clinical need, with a median albumin of 3.40 g/dL and 6.0% in-hospital mortality, and their reported threshold was correspondingly higher than ours (0.82 versus 0.555). One possible explanation for the different findings is the greater metabolic disturbance and event frequency in that cohort, so their result should not be read as transferable to an unselected elective population, and our null finding does not refute theirs. Our findings extend the question to a perioperative mixed-time-point ratio in elective surgery; they do not establish superiority over lactate or justify transfer of external cutoffs.

Associations between LAR and adverse outcomes have also been described in respiratory failure and sepsis [18], necrotizing soft-tissue infection [25], ischemic stroke and cerebral infarction [26,27], myocardial infarction [28], and heart failure [29]. Results are heterogeneous, and some adjusted analyses have not retained LAR as an independent marker. This variability supports context-specific validation and argues against treating LAR as a universal risk score.

A plausible interpretation is that preoperative albumin captures baseline inflammatory and physiological vulnerability, while immediate postoperative lactate captures the realized metabolic response to surgery. However, the ratio of measurements from different time points has not been established as a distinct biological construct. The exploratory spline showed no evidence of nonlinearity (*p* = 0.78), but sparse events and wide confidence intervals preclude strong conclusions about dose–response shape.

Higher LAR tertiles were associated with postoperative bleeding and longer intensive care stay, but these secondary analyses were exploratory and unadjusted for multiplicity. Because every patient entered intensive care by routine policy and stay was recorded in calendar days, a unit too coarse to separate a few hours from a full day, the small difference in stay across tertiles carries little interpretive weight. Immediate lactate may rise because of perioperative bleeding or hypoperfusion, creating reverse-causation or simultaneity concerns. These findings should not be interpreted as evidence that LAR causes complications.

Cutoff value and clinical application: The Youden threshold of 0.555 was derived from these data and is reported and analyzed at full precision, because the ratio is distributed narrowly enough that rounding alters the classification of individual patients. At this threshold, the positive predictive value was only 11.2%, and the negative predictive value of 97.6% was strongly influenced by the 6.0% mortality prevalence of the cohort. The threshold was selected and tested in the same cohort and should not guide treatment, discharge, or monitoring decisions without prospective external validation. At most, the present results support further study of LAR alongside clinical assessment and established risk factors.

### Limitations

This study has several limitations. It was retrospective and single-center with only 17 deaths, so the multivariable model is hypothesis-generating; ASA status, frailty, comorbidity burden, nutritional assessment, operative complexity, and intraoperative blood loss were unavailable and not imputed, leaving residual confounding. All resections were open, and no formal ERAS protocol was used, so the findings should not be extrapolated to minimally invasive resection or audited ERAS pathways. Complications were captured only as bleeding, anastomotic leak, and surgical site infection rather than Clavien–Dindo grades, and medical complications and cause of death were unrecorded, so morbidity is incompletely described. This gap is mainly descriptive, since leak and infection postdate the first-hour lactate and conditioning on such post-exposure events risks over-adjustment or collider bias. Bleeding is the exception: hemorrhage during or immediately after surgery can raise lactate and confound upstream, and because neither bleeding nor transfusion is time-stamped, we did not adjust for them and do not claim freedom from this confounding. Fluid, vasopressor, and transfusion given before the sample were likewise unrecorded; with the albumin denominator fixed preoperatively, these affect only the numerator, and their net direction is uncertain, since haemodilution and restored perfusion lower lactate whereas lactated crystalloids, adrenergic vasopressors, and stored blood raise it. The LAR literature has not isolated this exposure either: the cited MIMIC-IV analysis adjusted for vasoactive use within the first 24 h, a window that still straddles the sampled lactate [22]. No preoperative lactate or serial measurements were available, precluding preoperative LAR and trajectory analyses. The ratio did not outperform its components, secondary analyses were unadjusted for multiplicity, and the threshold was derived and tested internally, so bootstrap correction cannot replace external validation. Finally, the low event count reflects the observed mortality of an unselected consecutive cohort rather than case enrichment, and the high negative predictive value was driven by that low prevalence rather than discriminative advantage. Prospective multicenter studies with standardized sampling and detailed perioperative covariates are needed.

## 5. Conclusions

In conclusion, a perioperative ratio combining immediate postoperative lactate with preoperative albumin was associated with in-hospital mortality after age adjustment in this single-center cohort. Because both measurements are routinely available at no additional cost, LAR may offer a simple early signal for identifying patients who warrant closer postoperative monitoring. External validation in prospective multicenter cohorts is needed before these findings can inform clinical decision-making.

## Figures and Tables

**Table 1:** Baseline characteristics, perioperative factors, and clinical outcomes by LAR tertile.

Variable	Tertile 1 (*n* = 94)	Tertile 2 (*n* = 94)	Tertile 3 (*n* = 94)	*p*
Age, years, median [IQR]	68.5 [62.0–75.0]	70.5 [64.0–77.0]	72.5 [63.3–79.0]	0.09
Male sex, *n* (%)	51 (54.3)	62 (66.0)	63 (67.0)	0.13
Hypertension, *n* (%)	58 (61.7)	60 (63.8)	55 (58.5)	0.75
Diabetes mellitus, *n* (%)	23 (24.5)	33 (35.1)	28 (29.8)	0.28
Tumor localization, *n* (%)				0.16
Rectum	14 (14.9)	18 (19.1)	8 (8.5)	
Rectosigmoid	23 (24.5)	16 (17.0)	15 (16.0)	
Sigmoid	2 (2.1)	4 (4.3)	3 (3.2)	
Descending colon	13 (13.8)	10 (10.6)	22 (23.4)	
Transverse colon	0 (0.0)	1 (1.1)	0 (0.0)	
Right colon (ascending/cecum)	42 (44.7)	45 (47.9)	46 (48.9)	
Tumor stage, *n* (%)				0.70
Stage 0	5 (5.3)	3 (3.2)	4 (4.3)	
Stage I	14 (14.9)	14 (14.9)	14 (14.9)	
Stage II	34 (36.2)	32 (34.0)	34 (36.2)	
Stage III	37 (39.4)	37 (39.4)	30 (31.9)	
Stage IV	4 (4.3)	8 (8.5)	12 (12.8)	
Perioperative factors				
Neoadjuvant therapy, *n* (%)	4 (4.3)	9 (9.6)	7 (7.4)	0.36
Operation duration, min	140 [110–184]	150 [120–190]	145 [120–180]	0.31
Transfusion, *n* (%)	17 (18.1)	28 (29.8)	26 (27.7)	0.14
Postoperative outcomes				
In-hospital mortality, *n* (%)	2 (2.1)	5 (5.3)	10 (10.6)	**0.014 †**
Bleeding, *n* (%)	1 (1.1)	2 (2.1)	7 (7.4)	**0.018 †**
Anastomotic leak, *n* (%)	1 (1.1)	6 (6.4)	4 (4.3)	0.20
Surgical site infection, *n* (%)	9 (9.6)	11 (11.7)	5 (5.3)	0.29
ICU stay, days, median [IQR]	1 [1–2]	1 [1–2]	1 [1–3]	**0.009**
Total hospital stay, days	10.5 [8–14]	11 [8–14.8]	13 [8–16.8]	0.23
Preoperative laboratory measurements				
Hemoglobin, g/dL, mean ± SD	11.80 ± 2.00	11.51 ± 1.61	11.21 ± 1.97	0.10
WBC, ×10^3^/µL, median [IQR]	8.77 [6.45–11.79]	8.73 [7.08–12.26]	10.79 [7.89–14.06]	0.008
CRP, mg/L, median [IQR]	43.0 [11.3–136.3]	65.5 [28.0–122.8]	103.0 [35.0–170.5]	**0.003**
Creatinine, mg/dL, median [IQR]	0.80 [0.60–0.90]	0.80 [0.70–0.90]	0.80 [0.60–1.00]	0.49
Total protein, g/L, median [IQR]	62.0 [59.0–68.0]	61.0 [55.0–66.8]	55.5 [48.0–60.8]	**<0.001**
Preoperative albumin, g/dL, median [IQR]	3.70 [3.30–4.00]	3.50 [3.10–3.88]	3.00 [2.62–3.40]	**<0.001**
Immediate postoperative measurement and derived ratio				
Lactate, mmol/L, median [IQR]	1.22 [0.96–1.35]	1.74 [1.49–2.00]	2.60 [2.17–3.07]	**<0.001**
Perioperative LAR, (mmol/L)/(g/dL), median [IQR]	0.33 [0.30–0.37]	0.51 [0.46–0.55]	0.82 [0.71–0.99]	**<0.001**

Data are median [IQR], mean ± SD, or *n* (%). Continuous variables reported as median [IQR] were compared with the Kruskal-Wallis test and hemoglobin (mean ± SD) with one-way ANOVA, based on Shapiro-Wilk normality as-sessment; categorical variables were compared with the Pearson chi-square test, or with the Fisher-Freeman-Halton exact test for tumor localization and tumor stage owing to sparse cells. † Cochran-Armitage trend test across or-dered tertiles. LAR tertiles were defined empirically (*n* = 94 each; cutpoints 0.425 and 0.612) for descriptive com-parisons; continuous LAR was primary in regression. Albumin and all laboratory measures except lactate were preoperative; lactate was obtained within 1 h after intensive care unit admission, and LAR combined immediate postoperative lactate (mmol/L) with preoperative albumin (g/dL). Transfusion is listed among perioperative factors because its intraopera-tive-versus-postoperative timing was not recorded. All patients were admitted to intensive care postoperatively as routine policy; stay is counted in calendar days, so transfer to the ward before midnight on the day of surgery is recorded as zero days. Secondary comparisons were exploratory and unadjusted for multiplic-ity. WBC, white blood cells; CRP, C-reactive protein; LAR, lactate-to-albumin ratio; ICU, intensive care unit.

**Table 2:** Univariate and multivariable Firth’s penalized logistic regression for in-hospital mortality.

Variable	Univariate OR (95% CI)	*p*	Multivariable OR (95% CI)	*p*
LAR (per 0.1-unit increase)	1.17 (1.08–1.27)	**<0.001**	1.19 (1.09–1.29)	**<0.001**
Age (per year)	1.07 (1.01–1.14)	**0.014**	1.07 (1.01–1.14)	**0.014**
Tumor stage (III–IV vs. 0–II)	0.85 (0.32–2.24)	0.741	—	—
Neoadjuvant therapy	1.15 (0.20–6.51)	0.875	—	—
Diabetes mellitus	2.22 (0.85–5.80)	0.105	—	—
Hypertension	1.47 (0.52–4.13)	0.464	—	—
Sex (male)	1.07 (0.40–2.90)	0.887	—	—
High perioperative LAR (>0.5550505) ‡	4.71 (1.58–14.08)	0.006	4.27 (1.44–12.69)	0.009

OR, odds ratio; CI, confidence interval. Firth’s penalized logistic regression used Wald-based 95% CIs. The primary model was deliberately restricted to age and continuous LAR. The high-LAR estimate came from a separate exploratory age-adjusted model using the unrounded Youden threshold (>0.5550505); continuous and dichotomized LAR were not entered together. Fixed-model bootstrap validation used 1000 resamples, seed 2025, and no variable selection (apparent AUC 0.79; optimism-corrected AUC 0.78). The bold formatting in the table footer was used to emphasize the statistical significance. ‡ Dichotomized LAR was estimated in a separate age-adjusted model from continuous LAR, using the unrounded Youden threshold.

## Data Availability

The data presented in this study are available on request from the corresponding author.
